# A MEMS Variable Reluctance Sensor for Contactless Detection of a Ferrous Rotating Target

**DOI:** 10.3390/s26041280

**Published:** 2026-02-16

**Authors:** Dorra Nasr, Marco Baù, Alessandro Nastro, Stefano Bertelli, Marco Ferrari, Mohamed Hadj Said, Denis Flandre, Mounir Mansour, Fares Tounsi, Vittorio Ferrari

**Affiliations:** 1 Department of Information Engineering, University of Brescia, 25123 Brescia, Italy; dorra.nasr@unibs.it (D.N.); alessandro.nastro@unibs.it (A.N.); stefano.bertelli@unibs.it (S.B.); marco.ferrari@unibs.it (M.F.); vittorio.ferrari@unibs.it (V.F.); 2 Laboratory of Microelectronics and Instrumentation, Faculté des Sciences de Monastir, Université de Monastir, Monastir 5019, Tunisia; mounir.mansour@issatso.u-sousse.tn; 3 Centre for Research on Microelectronics and Nanotechnology of Sousse (CRMN), Technopole of Sousse B. P. 334, Sahloul, Sousse 4034, Tunisia; 4 SMALL Group, ICTEAM Institute, UCLouvain, 1348 Louvain-la-Neuve, Belgium; denis.flandre@uclouvain.be (D.F.); fares.tounsi@uclouvain.be (F.T.)

**Keywords:** micromachined coil, variable reluctance sensor, speed and proximity detection

## Abstract

Variable reluctance sensors are widely adopted for robust and contactless detection of motion in harsh and space-constrained environments. This paper presents a MEMS-based variable reluctance induction sensor for the noncontact characterization of rotating ferromagnetic targets, based on a micromachined planar micro-coil coupled with an external permanent magnet. The rotation of a ferromagnetic object modulates the magnetic circuit reluctance, generating a voltage signal across the micro-coil that encodes information on the target rotational speed, proximity, and cross-sectional shape. Sensor operation is investigated through a lumped-element magnetic–electrical circuit model and finite-element magnetostatic simulations, quantifying the effects of target diameter, distance, and angular position on the linked magnetic flux. Experimental validation is performed using rotating drill bits as representative targets and a dedicated high-gain, high-input-impedance front-end circuit to amplify the induced voltage. Measured results at fixed rotation frequency show periodic voltage waveforms whose amplitude and shape vary consistently with target geometry, proximity and speed. Reliable detection is achieved for rotational speeds up to 1500 rpm, for drill bit diameters as small as 5 mm, and at sensor-to-target distances up to 8 mm. These results demonstrate the potential of MEMS variable reluctance induction sensors for compact speed sensing and target shape detection.

## 1. Introduction

The rapid evolution of modern technology continues to drive the demand for compact, cost-effective, and high-performance sensors, particularly for applications requiring the measurement of position, rotational speed, and angular velocity [1]. Such sensing capabilities are essential in robotics, automotive systems, aerospace navigation, and various mechatronic platforms. Several sensing approaches have been employed to detect the motion of rotating targets, including magnetic [2], capacitive [3], ultrasonic [4], and optical techniques [5], each addressing specific requirements across a wide range of industrial and technological applications. Capacitive rotary sensors offer low power consumption and high sensitivity; however, their performance strongly depends on environmental conditions, and careful operation is required in the presence of dust, moisture, grease, or contamination, which can alter the dielectric properties of the sensing gap [6]. Optical angle sensors can provide very high resolution and are inherently immune to electromagnetic interference, but their accuracy and long-term reliability are significantly degraded in harsh environments due to sensitivity to dust, vibrations, misalignment, and optical path obstruction [7]. Ultrasonic sensing techniques enable noncontact distance and motion measurements and can operate with nonmetallic targets; nevertheless, they suffer from several limitations in rotating target applications, since their performances are highly dependent on the propagation medium and are affected by temperature gradients, airflow, humidity, and acoustic noise. In contrast, magnetic and inductive sensing techniques are largely insensitive to dust, moisture, grease, and oil, as these contaminants do not significantly affect magnetic fields. Consequently, provided that the target has specific properties, magnetic sensing solutions remain particularly attractive due to their noncontact operation, high reliability, robustness in harsh environments, and relatively low cost [8]. Their structural simplicity and compactness further support their widespread adoption in cost-sensitive and industrial applications requiring durable and maintenance-free sensing solutions.

Among magnetic sensing technologies, several solid-state solutions have been widely adopted due to their maturity and ease of integration. In particular, anisotropic magnetoresistive (AMR) sensors [9], giant magnetoresistive (GMR) sensors [10], tunneling magnetoresistive (TMR) sensors [11], and Hall-effect sensors [12] are commonly employed for position, speed, and field measurements. While these sensors provide high sensitivity and compact form factors, they typically require active biasing, signal conditioning, and precise magnetic field control, which may limit their suitability in high-temperature, high-vibration, or power-constrained environments. In this context, variable reluctance (VR) sensors constitute a well-established and robust class of magnetic sensors used across multiple application domains, offering effective measurement of parameters such as rotational speed [13] and angular position [14] of conductive or ferromagnetic components. Conventional VR sensors generally consist of a permanent magnet and one or more coils forming a magnetic circuit whose reluctance varies with the position of a nearby target. While the target static position can be sensed by measuring the coil inductance, VR induction sensors, most commonly referred to as VR sensors, as in the present paper, detect target motion [15]. Their operating principle relies on the transduction of time-varying magnetic flux into an electromotive force (EMF) according to Faraday’s law of induction [16]. VR sensors require no external power source for operation, except for optional signal amplification. Consequently, these attributes make VR sensors suitable for a variety of applications, including industrial automation [17,18], electric vehicles (EVs) motor control [19,20], and as mechano-electrical converters in energy harvesting [21].

Recent advancements in Micro-Electro-Mechanical Systems (MEMS) technology have stimulated growing interest in miniaturized coil-based sensors. MEMS implementations promise unique advantages, such as high spatial resolution, improved sensitivity, batch manufacturability, and seamless integration with low-power electronics [22]. While inductive MEMS sensors have been reported for accelerometers [23,24], microparticle detection [25], and displacement sensing [26], only a limited number of MEMS-scale VR devices have been documented to date. Magnetic field [27,28] and micropositioning [29] VR microsensors have been proposed, and a micro variable reluctance sensor (µVRS) has demonstrated the feasibility of detecting a rotating ferromagnetic gear ring with adequate signal-to-noise ratio [30]. Preliminary results leading to the development of the MEMS VR sensor presented in this work have been recently reported in [31]. Further investigations are required to understand how microscale geometries, magnetic circuit constraints, and target characteristics influence the performance of miniaturized VR sensors. In particular, the interaction between a MEMS micro-coil, an external permanent magnet, and a rotating ferromagnetic object remains largely unexplored, and systematic analyses combining analytical modeling, finite-element simulations, and experimental validation appear to be lacking.

In this context, this work presents the design, modeling, fabrication, and experimental characterization of a MEMS-based micro-variable-reluctance sensor intended for detecting the rotation of ferrous drill bits. The proposed device employs a micromachined planar micro-coil coupled to a permanent magnet, forming a compact magnetic circuit whose reluctance is periodically modulated by the rotating target. The micro-coil is fabricated using MEMS-compatible processes, enabling high reproducibility and potential for future integration into fully miniaturized sensing modules. A combination of analytical modeling, lumped-element magnetic–electrical equivalent circuits, and finite-element simulations is used to describe the sensor functioning and analyze the influence of geometrical and operational parameters. Experimental results obtained using a microsensor prototype in front of a rotating drill bit validate the theoretical predictions.

The remainder of this paper is organized as follows. Section 2 introduces the operating principle of the proposed VR microsensor. Section 3 presents the finite-element-method (FEM) simulations, in which a stationary study is performed to evaluate the sensor operation under different conditions. Section 4 details the MEMS fabrication process, experimental setup, and measurement results. Section 5 provides concluding remarks.

## 2. Working Principle

**Figure 1:**
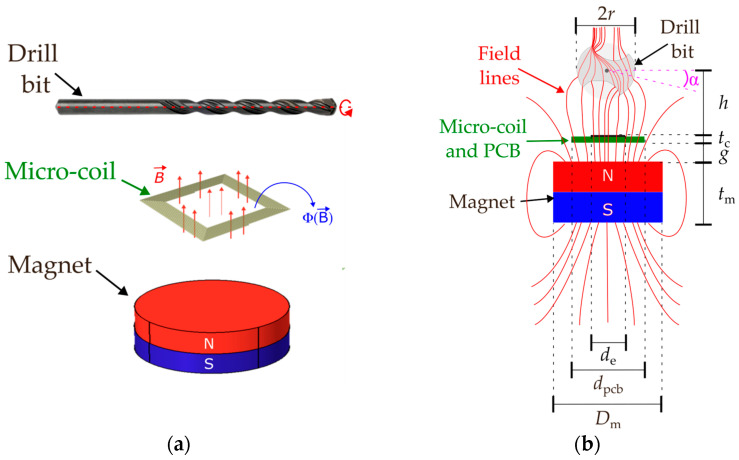
Working principle of the proposed variable reluctance sensor, including the relevant geometrical parameters: (**a**) overview of the experimental configuration and main elements of the sensing setup, and (**b**) cross-sectional schematic illustrating how the rotating ferromagnetic drill bit deflects the magnetic field lines, thereby modulating the reluctance of the magnetic circuit formed by the permanent magnet, the air gap, and the drill bit.

The proposed VR microsensor consists of a square planar micro-coil placed within the static magnetic field $\vec{B}$ generated by an axially magnetized cylindrical permanent magnet. A ferromagnetic drill bit rotating about its longitudinal axis acts as the target, whose periodic motion perturbs the magnetic circuit, as illustrated in Figure 1a. A cross-sectional view of the system, including the relevant geometrical parameters, is shown in Figure 1b. The drill bit has diameter 2*r*, while the permanent magnet has diameter *D*_m_ and height *t*_m_. The squared planar micro-coil, with outer side length *d*_e_, is mounted on a square printed circuit board (PCB) with side length *d*_pcb_, resulting in an overall stack height *t*_c_. The PCB–coil assembly is placed at a separation distance *g* from the top surface of the magnet, and the upper surface of the micro-coil is located at a distance *h* from the rotation axis of the drill bit. When the drill bit rotates, its helicoidal two-flute cross-sectional geometry deflects the magnetic field lines and thus modulates the magnetic reluctance of the circuit formed by the permanent magnet, the air gap, and the drill bit itself. Magnetic reluctance represents the opposition of a magnetic path to the flow of magnetic flux; therefore, time-varying reluctance produces a corresponding variation in the magnetic flux linked to the micro-coil. According to Faraday’s law, this time-varying flux induces a voltage across the coil. In the investigated configuration, the adoption of a square coil over other geometries has the inherent advantage of maximizing the area coverage on the die and thus the linked flux. The rotation angle of the drill bit with respect to the micro-coil is denoted by α. In variable reluctance sensors, the induced voltage constitutes the primary output quantity, with its amplitude and frequency directly influenced by the target geometry, proximity, and angular velocity [17,18].

A simplified analytical model of the proposed sensor can be formulated by representing the permanent magnet through its equivalent magnetomotive force (MMF), denoted as $F_{m}$, and by modeling the magnetic path using lumped reluctances. In particular, $R_{m}$, $R_{air}$ and $R_{bit}$ denote the magnetic reluctances of the permanent magnet, the air gap, and the ferromagnetic drill bit, respectively. Under the assumption that the magnetic flux density is uniformly distributed and equally linked across the effective number of turns *N*_eff_ of the micro-coil, the magnetic flux $\Phi_{B}$ coupled to the coil can be expressed as [32]:

(1) $$\Phi_{B}=\frac{N_{eff}\;F_{m}}{R_{m}+R_{air}+R_{bit}},$$

Since the majority of the magnetic path lies in air and the drill bit is characterized by a high magnetic permeability, the total reluctance of the circuit is dominated by $R_{air}$ + $R_{m}$. Consequently, it can be assumed that $R_{air}$ + $R_{m}$ >> $R_{bit}$ and Equation (1) can be expanded to the first order with respect to $R_{bit}/(R_{air}$ + $R_{m})$, yielding:

(2) $$\Phi_{B}\approx \frac{N_{eff}F_{m}}{R_{m}+R_{air}}\left(1-\frac{R_{bit}}{R_{m}+R_{air}}\right).$$

As the drill bit rotates with angular velocity ω=dα/dt then, by considering the cross section of the drill bit in Figure 1b, the magnetic reluctance associated with its cross section $R_{bit}(t)$ varies periodically. Owing to the bit two-flute geometry, the reluctance can be approximated as:

(3) $$R_{bit}(t)=R_{0}+∆R\sin\left(2\omega t\right),$$

where $R_{0}$ represents the reluctance of the central cylindrical core of the drill bit, while ΔR accounts for the modulation introduced by the helicoidal flutes. The factor 2ω reflects the presence of two dominant geometrical features per revolution. Both Equations (2) and (3) do not account for the actual geometry of the cross section of the drill bit and neglect that during the revolution of the drill bit also $R_{air}$ slightly changes over time. By substituting Equation (3) into (2) and applying Faraday’s law, the voltage *v*_s_(*t*) induced across the micro-coil can be expressed as:

(4) $$v_{s}\left(t\right)=-\frac{d\Phi_{B}}{dt}=\frac{N_{eff}F_{m}}{{\left(R_{m}+R_{air}\right)}^{2}}\;\frac{dR_{bit}}{dt}=\frac{2\omega N_{eff}F_{m}\Delta R}{{\left(R_{m}+R_{air}\right)}^{2}}\cos\left(2\omega t\right).$$

Equation (4) highlights that (*i*) the induced voltage *v*_s_(*t*) exhibits a frequency that is twice the rotational frequency of the drill bit (as a direct consequence of its two-flute geometry); and (*ii*) the voltage amplitude scales linearly with the rotation speed, the magnet strength $F_{m}$, and the effective number of turns *N*_eff_ of the micro-coil. In particular, the product $N_{eff}F_{m}$ suggests that at parity of *v*_s_(*t*). amplitude, a tradeoff between the number of turns of the coil and the strength of the magnet can be exploited to tune the dimensions of the sensor, since both *N*_eff_ and $F_{m}$ directly relate to the dimensions of the coil and magnet, respectively.

**Figure 2:**
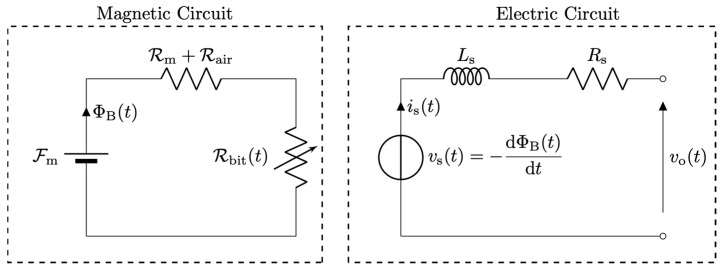
L Equivalent lumped-element circuit of the VR sensor for *i*_s_(*t*) = 0. The magnetic circuit includes a time-varying reluctance $R_{bit}(t)$ to model the rotating drill bit (Equation (3)). The electrical circuit is coupled to the magnetic domain through the controlled voltage source *v*_s_(*t*), which represents the voltage induced in the micro-coil according to Equation (4).

Equations (1)–(4) directly lead to the lumped-element magnetic–electrical equivalent circuit, shown in Figure 2. The circuit consists of a magnetic domain and an electrical domain, formulated using the classical magnetic–electric analogy. In the magnetic part, the voltage source represents the magnetomotive force $F_{m}$ generated by the permanent magnet, the current represents the magnetic flux $\Phi_{B}$, and the resistive elements $R_{m}$, $R_{air}$ and $R_{bit}\left(t\right)$ model the magnetic reluctances of the magnet, the air gap, and the ferromagnetic drill bit, respectively. The reluctance $R_{bit}\left(t\right)$ is explicitly time dependent, as it accounts for the periodic modulation of the magnetic circuit caused by the rotation of the drill bit, as described by Equation (3). The magnetic and electrical domains are coupled through a controlled voltage source *v*_s_(*t*), which represents the voltage induced across the micro-coil as a consequence of the time variation of the magnetic flux, in accordance with Faraday’s law and Equation (4). The electrical behavior of the micro-coil is modeled by its series resistance *R*_s_ and inductance *L*_s_. In principle, a more refined model could account for the dependence of *L*_s_ on the instantaneous magnetic reluctance $R_{bit}(t)$, as well as for the counteracting magnetomotive force produced by the coil current *i*_s_(*t*).

However, when the sensor output is read under open-circuit conditions or through a high-input-impedance front-end, the coil current satisfies *i*_s_(*t*) ≈ 0, rendering both effects negligible. Under these conditions, the measurable output voltage coincides with the induced voltage, i.e., *v*_o_(*t*) = *v*_s_(*t*).

## 3. Results of FEM Analysis

**Figure 3:**
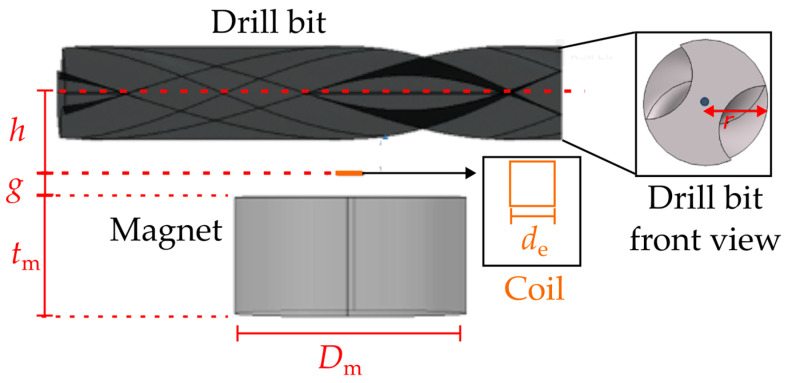
Close-up view of the simulated geometry, highlighting the equivalent one-turn coil and the drill bit cross section.

A finite-element model (FEM) was developed in COMSOL Multiphysics (ver 6.0) to analyze and validate the operating principle of the proposed VR sensor under various geometrical and operational conditions. To reduce computational complexity and simulation time, several simplifying assumptions were introduced. In particular, the actual micromachined multi-turn planar coil was replaced with an equivalent single-turn square loop having the same outer side length, *d*_e_ = 2180 µm, as shown in Figure 3. The permanent magnet was modeled as a uniformly magnetized neodymium cylindrical magnet with relative permeability μ_r_ = 1.05, diameter *D*_m_ = 20 mm, height *t*_m_ = 10 mm, and remanent flux density *B*_r_ = 1.2 T. The drill bit was modeled as an iron target with relative permeability μ_r_ = 4000, accurately reproducing its helicoidal two-flute cross-section geometry. The entire structure was enclosed within a sufficiently large spherical air domain to ensure accurate computation of the magnetic field decay and to minimize boundary effects. Magnetostatic simulations were then performed to compute the magnetic flux density $\vec{B}$ throughout all domains. The magnetic flux Φ_B_ linked to the simplified one-turn coil was obtained by integrating the normal component of $\vec{B}$ over the loop area. The simulations iteratively evaluated the variation of Φ_B_ as a function of the drill bit angular position α over a full mechanical revolution. In addition, parametric analyses were carried out to investigate the influence of key geometric parameters, namely, the bit–coil vertical distance *h*, the drill bit radius *r*, and the magnet diameter *D*_m_. Based on the simulated flux data Φ_B_(α), and assuming a constant angular velocity, the corresponding induced voltage *v*_s_(*t*) was obtained through numerical differentiation in post-processing.

**Figure 4:**
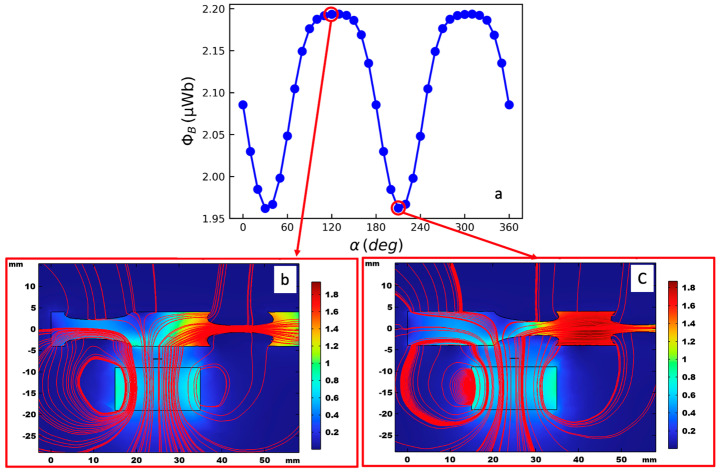
Magnetic flux linked to the coil as a function of the drill bit rotation angle α: (**a**) Distribution of the magnitude (color map) and field lines of the magnetic flux density $\vec{B}\;$ for α = 110° (**b**) and α = 200° (**c**), shown at the central cross-section of the geometry.

Figure 4a shows simulated magnetic flux Φ_B_ linked to the coil for a full revolution of the drill bit (α from 0° to 360°) for the representative case *r* = 4 mm, *g* = 2 mm and *h* = 9 mm. Compared with the predictions of Equations (2) and (3), the waveform is not strictly sinusoidal, due to the fact that the magnetic field is nonuniform around and inside the drill bit. However, as expected, the two-flute geometry of the drill bit produces two maxima and two minima of Φ_B_(α) per revolution. Figure 4b,c illustrate the distributions of the magnetic flux density magnitude (color map) and the associated field lines for the angular positions α = 110° and α = 200°, respectively, shown at the central cross-section of the simulated geometry. For a given angle position, the magnitude of Φ_B_ is directly related to the effective extension of the ferromagnetic material above the coil along its normal axis. This extension is maximal at α = 110° and minimal for α = 200°, corresponding to the maximum and minimum values of Φ_B_, respectively. When α is mapped to time, these flux variations translate directly into the induced voltage waveform at the coil terminals. Specifically, for the configuration of Figure 4a, the peak-to-peak variation of Φ_B_ is approximately $\Delta \Phi_{B}\approx 0.23\;\mu Wb$, and if the drill bit is rotating at constant angular rate ω=50π rad/s, corresponding to 1500 rpm (revolutions per minute), then the resulting induced voltage $v_{s}$ for the considered single-turn coil is about 36 μV.

**Figure 5:**
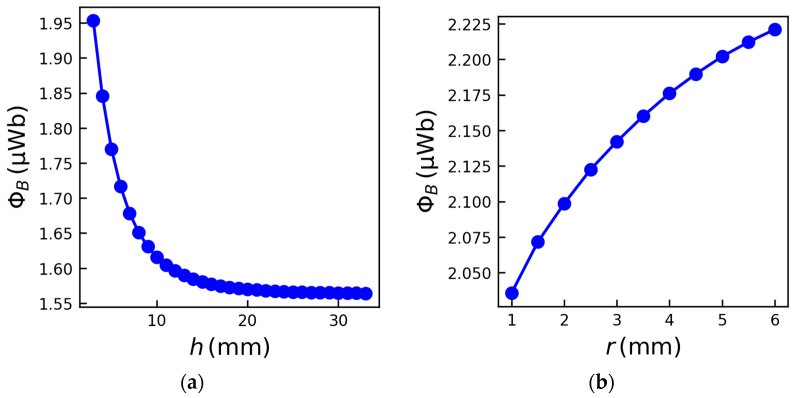
FEM simulation results of Φ_B_ as a function of (**a**) the distance *h* for *r* = 4 mm, *g* = 2 mm and α = 110°, and (**b**) the drill bit radius *r* for *g* = 2 mm, *h* − *r* = 3 mm and α = 110°.

To further assess the impact of target geometry on sensor performance, additional parametric studies were carried out. Figure 5a shows the variation of Φ_B_ as a function of the coil–target distance *h*, which was varied from 3 to 35 mm in increments of 1 mm while keeping all other parameters constant. As *h* increases, Φ_B_ decreases monotonically and eventually converges to approximately 1.55 μWb, i.e., the residual flux due to the magnet only, and indicates that beyond a certain distance, the presence of the ferromagnetic target no longer significantly perturbs the magnetic field distribution. Next, the influence of the drill bit radius *r* was investigated by keeping the distance from the drill bit edge constant at (*h* − *r*) = 3 mm. The results, shown in Figure 5b, demonstrate that Φ_B_ increases with increasing *r*. This behavior is expected, as a larger ferromagnetic cross-section intercepts and redirects a larger fraction of the magnetic field lines toward the coil, thereby increasing the linked magnetic flux.

**Figure 6:**
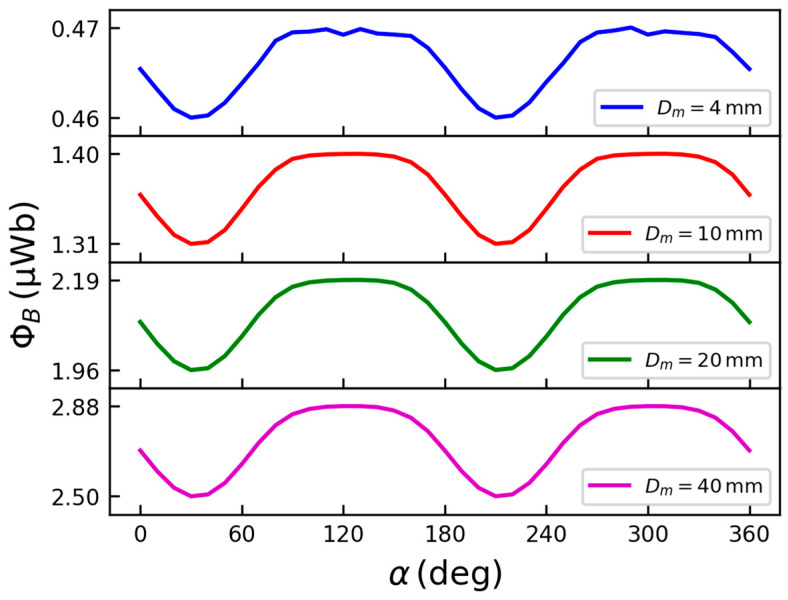
FEM simulation results of the flux Φ_B_ as a function of the drill bit rotation angle α for different magnet diameters *D*_m_.

Finally, the effect of the magnet diameter *D*_m_ was also studied. Figure 6 reports the simulated Φ_B_(α) for four different values of *D*_m_. For each case, the magnet height was set to *t*_m_ = *D*_m_/2, while the remanent flux density was kept constant at *B*_r_= 1.2 T. It can be observed that increasing *D*_m_ leads to a corresponding increase in Φ_B_. This behavior can be attributed to the larger magnet producing a spatial distribution of the magnetic field with a higher normal component across the coil surface, thereby enhancing the linked flux and the expected induced voltage. Conversely, when *D*_m_ = 4 mm, the magnet dimensions become comparable to those of the coil, causing the field lines to bend more strongly toward the opposite magnetic pole. As a result, the in-plane component of the magnetic field dominates, leading to a reduction in the flux linked to the coil.

## 4. Experimental Activities and Results

### 4.1. Design, Fabrication and Experimental Setup

**Figure 7:**
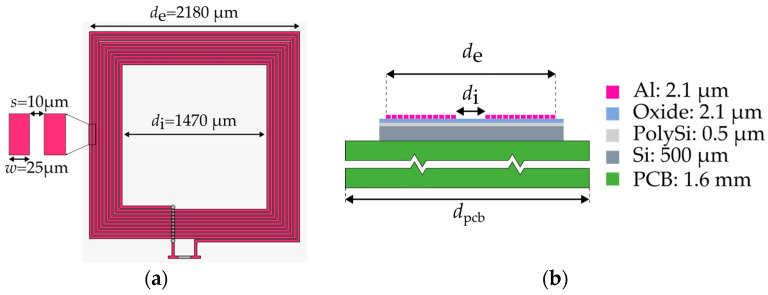
Top view of the micromachined micro-coil with relevant geometrical dimensions (**a**). Layer stack of the adopted MEMS fabrication process (**b**).

Figure 7a shows the layout and main geometrical parameters of the MEMS micro-coil used for experimental validation. The device consists of a square planar spiral with *N* = 11 turns, an outer side length *d*_e_ = 2180 µm, an inner side length *d*_i_ = 1470 µm, a conductor width *w* = 25 µm, and an inter-turn spacing *s* = 10 µm. Figure 7b illustrates the layer stack adopted in the fabrication process, which comprises four photolithographic steps. The micro-coil was fabricated on a 500 µm-thick trap-rich, high-resistivity silicon substrate, integrated with a 0.5 µm-thick polysilicon layer, a 2.1 µm-thick silicon oxide insulating layer, and a 2 µm-thick aluminum top metallization. The use of a trap-rich substrate minimizes charge leakage into the silicon bulk, thereby improving the inductor quality factor and reducing substrate-related losses [33]. After fabrication, the micro-coil chip was wire-bonded onto a 1.6 mm-thick printed circuit board (PCB) with side length *d*_pcb_ = 2 cm, enabling reliable electrical interfacing with the readout electronics.

**Figure 8:**
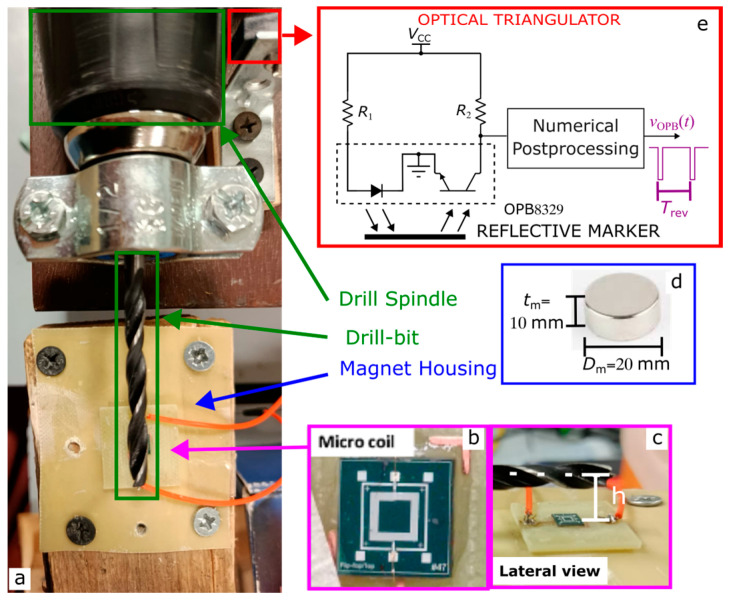
Experimental setup (**a**). Close-up view of the micro-coil (**b**) and corresponding lateral view (**c**). The wooden support holds inside the magnet (**d**). Optical sensor used for monitoring the spindle revolution (**e**).

The experimental setup used to validate the sensor operation is shown in Figure 8a. A ferromagnetic drill bit was mounted on a motorized drill spindle and positioned at a controlled distance *h* from the micro-coil, as detailed in Figure 8b,c. The PCB carrying the micro-coil was mounted on a wooden support that housed a cylindrical N42-grade neodymium permanent magnet with diameter *D*_m_ = 20 mm, height *t*_m_ = 10 mm, and nominal remanent flux density *B*ᵣ = 1.2 T, as shown in Figure 8d. The vertical separation between the magnet and the top surface of the micro-coil was fixed at *g* = 2 mm. To accurately adjust the coil–target distance *h,* the magnet housing and PCB assembly were mounted on a micrometric linear translation stage. The rotational speed of the drill bit was independently monitored using an OPB8329 (Optek, Essen, Germany) optical sensor comprising a photodiode–phototransistor pair, positioned close to the drill spindle as shown in Figure 8e. A reflective marker attached to the spindle generates one optical pulse per mechanical revolution, allowing precise extraction of both the angular position reference signal *v*_opb_(*t*) and the corresponding revolution period *T*_rev_.

### 4.2. Front-End Electronics

**Figure 9:**
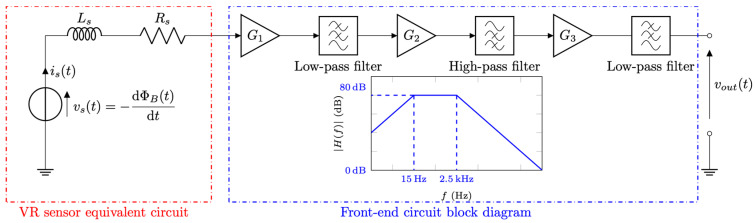
Electrical equivalent circuit of the VR sensor and block diagram of the front-end circuit used for low-noise voltage amplification. The inset shows the asymptotic frequency response of the amplifier.

Figure 9 shows the block diagram of the electronic front-end circuit designed to extract and amplify the signal generated by the VR microsensor. As discussed in Section 2, the electrical model of the micro-coil consists of its series resistance *R*_s_ and inductance *L*_s_, together with the induced voltage source *v*_s_(*t*) = −dΦ_B_/d*t* accounting for the time-varying magnetic flux linked to the coil. The intrinsic electrical parameters of the micro-coil were measured at 100 kHz using an impedance analyzer, yielding *R*_s_ = 69 Ω and *L*_s_ = 82 nH. To ensure negligible coil current, i.e., *i*_s_(*t*) = 0, the sensor output was amplified using a high-input-impedance, three-stage voltage amplifier with an overall gain *G* = *G*_1_*G*_2_*G*_3_ ≅ 10^4^, corresponding to nearly 80 dB. For the board-level prototype, bipolar-junction transistor (BJT) operational amplifiers with 1 MHz gain-bandwidth product were employed. First-order high-pass and low-pass filtering stages were included to suppress DC offsets and reject out-of-band noise. The resulting band-pass frequency response, schematically illustrated in the inset of Figure 9, has −3 dB cutoff frequencies at 15 Hz and 2.5 kHz, which are adequate for the expected frequency range of the VR sensor signal.

### 4.3. Results and Discussion

**Figure 10:**
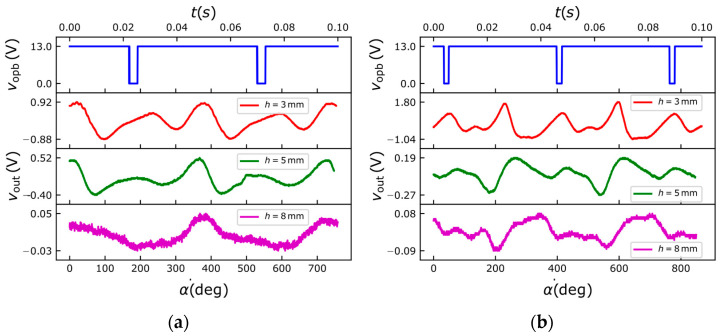
Measured *v*_out_ and *v*_opb_ signals as a function of the revolution angle α and time *t* taken at the distances *h* = 3 mm, *h* = 5 mm and *h* = 8 mm for drill bits with (**a**) *r* = 2.5 mm and (**b**) *r* = 4 mm.

To experimentally validate the proposed VR microsensor, measurements were performed using two ferromagnetic drill bits with different radii, namely *r* = 2.5 mm and *r* = 4 mm. For each drill bit, the amplified sensor output voltage *v*_out_(*t*) and the optical reference signal *v*_opb_(*t*) were simultaneously recorded at three different coil–target distances: *h* = 3 mm, *h* = 5 mm, and *h* = 8 mm. Figure 10a,b show the measured waveforms *v*_out_(*t*) and *v*_opb_(*t*) as a function of the rotation angle α and time *t* for the two drill bit radii. The optical reference signal indicates revolution periods of *T*_rev_ = 48 ms and *T*_rev_ = 42 ms, corresponding to rotational speeds of about 1250 rpm and 1429 rpm, respectively. By analyzing the signals, it can be observed that for each complete revolution, identified by the time between two consecutive pulses in *v*_opb_(*t*), two peaks and two valleys are present in *v*_out_(*t*). This behavior is consistent with the two-flute geometry of the drill bits and closely matches the predictions obtained from both the analytical model and FEM simulations. Specifically, the measured output voltage *v*_out_ reflects the time variation in the shape of the cross section of the target along the vertical axis, averaged across the coil surface. However, the waveform within each period is less regular with respect to the simulation results of Figure 6, most likely due to not perfect parallelism between the coil and the drill bit and residual oscillations of the drill bit clamped in the spindle. Additionally, peaks and valleys in *v*_out_(*t*) do not always occur at the same time with respect to the pulses in *v*_opb_(*t*) due to the different initial angular position of the drill bit in the spindle with respect to the reflective marker, resulting in a varying time offset throughout the different tests. Moreover, the rotational speed can be accurately extracted from the temporal spacing between successive peaks or valleys in the sensor output. As the coil–target distance *h* increases, a progressive reduction in the peak-to-peak amplitude of *v*_out_ is observed, in agreement with the simulation results. In particular, for *h* = 8 mm, a noticeable decrement of the signal-to-noise ratio occurs, although the periodic signature of the rotation remains clearly detectable. For the case *h* = 3 mm and *r* = 4 mm, the maximum output voltage variation is approximately $\left|\Delta v_{out}\right|$ = 2.8 V, corresponding to an induced micro-coil voltage of $\left|\Delta v_{s}\right|\approx \left|\Delta v_{out}\right|/G=280\mu V$. These results confirm that the proposed MEMS-based VR microsensor is capable of reliably detecting both the rotational speed and the geometric characteristics of ferromagnetic targets, even when operating at millimeter-scale stand-off distances.

## 5. Conclusions

This paper presented the design, modeling, fabrication, and experimental validation of a variable reluctance microsensor based on a micromachined planar micro-coil combined with an external cylindrical permanent magnet. When a ferromagnetic object forms part of the magnetic circuit, time variations in magnetic reluctance are transduced into an induced voltage across the micro-coil. A rotating ferromagnetic drill bit was used as the target to demonstrate the sensing principle and evaluate the sensor performance. The proposed microsensor was investigated using a combined theoretical and numerical approach. A simplified lumped-element equivalent circuit was developed to describe the operating principle and to relate the induced voltage to the time-varying reluctance of the magnetic circuit. Finite-element simulations performed in COMSOL Multiphysics provided detailed insight into the magnetic flux distribution and quantified the influence of key geometric parameters, including magnet dimensions, drill bit diameter, and sensor-to-target distance. The simulation results confirmed the expected periodic modulation of the magnetic flux and its dependence on target geometry and proximity. Experimental validation was carried out using a purposely fabricated MEMS micro-coil connected to a tailored high-gain, high-input-impedance front-end circuit. Due to the microvolt-level amplitude of the sensor signal, the front-end circuit was specifically designed to provide sufficient amplification while rejecting out-of-band noise. The experimental results showed good agreement with the theoretical predictions and FEM simulations. Reliable detection of target rotation speed and geometric features was achieved for sensor-to-target distances of up to 8 mm with an adequate signal-to-noise ratio. These results demonstrate the feasibility of the proposed MEMS variable reluctance microsensor and highlight its potential for compact noncontact sensing of rotation and shape features of small ferromagnetic components in industrial and precision monitoring applications.

## Data Availability

Dataset available on request from the authors.
